# *Languages of Grief*: a model for understanding the expressions of the bereaved

**DOI:** 10.1080/21642850.2013.879041

**Published:** 2014-01-22

**Authors:** Inge B. Corless, Rana Limbo, Regina Szylit Bousso, Robert L. Wrenn, David Head, Norelle Lickiss, Hannelore Wass

**Affiliations:** ^a^ MGH Institute of Health Professions, Boston, MA02116, USA; ^b^ Gundersen Health System, La Crosse, WI, USA; ^c^ School of Nursing, University of São Paulo, São Paulo, Brazil; ^d^ Department of Psychology, University of Arizona, Tucson, AZ, USA; ^e^ Flebbs Benefice, Anglican Diocese of Norwich, UK; ^f^ Royal Prince Alfred and Royal Hospital for Women, University of Sydney, Sydney, Australia; ^g^ College of Education, University of Florida, Gainesville, FL, USA

**Keywords:** bereavement, communication, grief, language, professional

## Abstract

The aim of this work is to provide an overview of the key features of the expressions of grief. Grief is a response to loss or anticipated loss. Although universal, its oral and nonverbal expression varies across cultures and individuals. Loss is produced by an event perceived to be negative to varying degrees by the individuals involved and has the potential to trigger long-term changes in a person's cognitions and relationships. The languages used by the bereaved to express grief differ from the language used by professionals, creating dissonance between the two. Data were obtained from English language Medline and CINAHL databases, from professional and personal experiences, interviews with experts, and exploration of cemetery memorials. Blog websites and social networks provided additional materials for further refinement of the model. Content analysis of the materials and agreement by the authors as to the themes resulted in the development of the model. To bridge the gap between professional language and that used by the bereaved, a *Languages of Grief* model was developed consisting of four *Modes* of *Expression,* four *Types of Language,* plus three *Contingent Factors.* The *Languages of Grief* provides a framework for comprehending the grief of the individual, contributing to clinical understanding, and fruitful exploration by professionals in better understanding the use of languages by the bereaved. Attention to the Modes of Expression, Types of Language, and Contingent Factors provides the professional with a richer understanding of the grieving individual, a step in providing appropriate support to the bereaved. The *Languages of Grief* provides a framework for application to discrete occurrences with the goal of understanding grief from the perspective of the bereaved.

## 1. Introduction

Grief is universal. Its oral and nonverbal expression varies across cultures as well as individuals, and is a response to loss or anticipated loss. Grief and the expression of grief is an articulation not only of loss but potentially of gain, growth, and the birth pangs of a new personal synthesis. The *Languages of Grief* used by the bereaved differ from the language utilized by professionals, creating dissonance between the two.

Professionals develop an in-depth understanding of the meaning of the scientific language they use, or at least knowledge about an argot that is both shorthand for scientific communication and a symbol of professional membership. It also serves as a distancing mechanism from the pain of the griever. With the changes in our social construction of death, dying, and bereavement, and particularly of grief being “time-bounded” (Fulton, 2003, p. 347), there have been changes in the nomenclature to describe such events including anticipatory grief, disenfranchised grief, prolonged grief disorder, and complicated grief (Shear et al., 2007). Even so, the terms grief and bereavement are sometimes used interchangeably.

Grief is usually described as a “normal, healthy, healing and ultimately transforming response to a significant loss that usually does not require professional help, although it does require ways to heal the broken strands of life and to affirm existing ones” (Schneider, 2000, p. 7). Some of the major influences on the literature and the language used to discuss grief include psychoanalytic theory (Freud, 1953, 1963); the focus on grief in response to an acute event (Lindemann, 1944); stages of grief (Kubler-Ross, 1969); grief in response to a major life transition (Parkes, 1970; Parkes & Weiss, 1983); attachment theory (Bowlby, 1982); grief as a response to a stressful life event (Stroebe & Stroebe, 1987); and the formulation of two-track model of grief (Rubin, 1981; Rubin, Malkinson, & Witztum, 2012) to mention a few of the prominent theorists writing on the subject of grief.

In addition to the literature on the process of grieving, bereavement and its expression are also of considerable interest to scholars. Bereavement as the result of the death of a loved one is a socially constructed status with both personal and societal meaning. For example, black-edged stationery was used in Victorian times as a sign of the bereaved status of the writer. The wearing of black clothing by widows for a year conforms to societal expectations in some cultures, yet also may be an outward expression of personal grieving. Bereavement from a societal perspective entails specified behaviors on the part of those who are bereaved, as well as those who interact with the bereaved.

Professional language is a means of synthesizing theoretical formulations and observed phenomena about the process of grieving into a vocabulary useful for professional discourse. For example, manifestations of grief have been categorized as physical, cognitive, emotional, and behavioral (Corless, 2010; Corless, Cartier, & Guarino, 2011; Doka, 1989). One of the unintended consequences of the use of such language by professionals with the bereaved about the process of grieving is that it may create a barrier to communication by distancing the bereaved from their expressions of grief. Furthermore, when the language of the process of grieving is appropriated by the bereaved, the meanings and emotions expressed in the language of grief used by the bereaved may become lost in the translation. A more helpful approach to the griever may be to focus on the personal languages used by the bereaved in their expressions of grief. Expressions of grieving can be distinguished from the process of grieving and the state of bereavement. And it is the expressions of grief that are the focus of this paper.

## 2. Background

The literature is rich concerning the process of grieving with conceptualizations that range from the goal of “closure” for the grieving person to the more recent acknowledgement of “continuing bonds”; namely, that the death of a person who is important to the individual remains a loss although the individual accommodates to the absence of the individual in varying ways and to varying degrees. It is important to note “there is no one right or universal way to experience or respond to loss” (Rubin et al., 2012, p. 20). As will be evident, the authors of this paper give no precedence to any one type of response in their discussion of the *Languages of Grief*. Rather the model of the *Languages of Grief* is a codification of the expressions of grief.

Silverman (2007) noted “The vocabulary we use to describe and explain the experience [of grief] may not be consistent with the experience of the bereaved” (p. 168). Health-care professionals have utilized a professional vocabulary to describe the bereaved according to various phases in the grieving process. Physicians, nurses, psychologists, social workers, and other professionals share in using these frameworks. Such an approach does not consider the expressions of grieving per se and leaves the bereaved in the uncomfortable situation of having to fit into the frameworks used by professionals. An approach where professionals attend to the expressions of the bereaved may be more helpful to those who are grieving. It is for this reason that we investigated the manifestations of grief and developed the model of *Languages of Grief*, an approach to thinking about the ways that grief is experienced, represented, and expressed.

In this paper, we focus on four Modes of Expression and four Types of Language. The Modes of Expression capture the manner in which grief is indicated. The Types of Language consist of the representational approaches to expressing grief. We also identify three categories of Contingent Factors that influence how an individual processes grief. The Contingent Factors are historical, personal, social, and cultural conditions that influence the expression of grief. The model of *Languages of Grief* provides a framework for considering the varied manifestations of grief as expressed by the griever. For this schema, we use the plural term “languages” to demonstrate the multiple layers, contexts, and categories that provide a framework for the complexities and nuances the concept offers. The *Languages of Grief* are a highly personal and subjective set of ways to express or withhold emotions as responses to real, perceived, or anticipated losses in different contexts.

## 3. Methodology

Data were derived from the English language scientific literature using Medline and CINAHL databases with the keywords: language, grief, communication, and expression, and from professional sources including face-to-face interviews with internationally known experts in the field. Finally, exploration of cemetery memorials in three different countries and investigation of virtual memorials on the Internet provided additional data. Using content analysis, information was obtained, processed, and concepts identified, enriching our understanding of the *Languages of Grief*.

## 4. The vocabulary of the *Languages of Grief*

**Figure 1.**
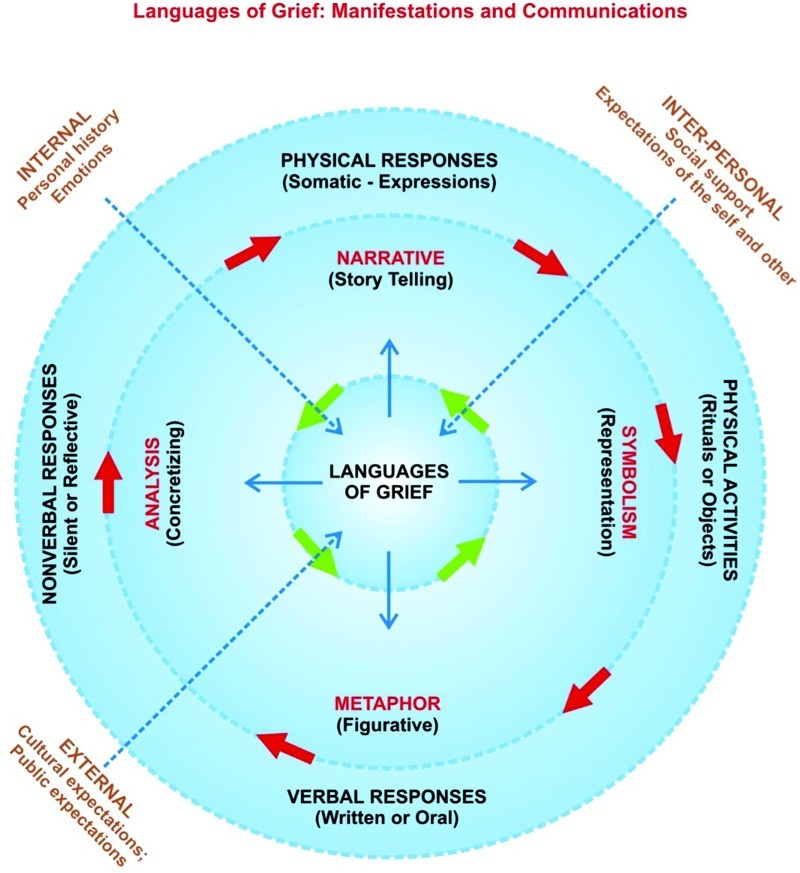
*Languages of Grief*: manifestations and communications.

The Modes of Expression and Types of Language constitute the infrastructure of our schema of the *Languages of Grief*. These two elements are combined in various ways by the bereaved, either consciously or unconsciously, to manifest and express their use of the *Languages of Grief*. Further, Modes of Expression and Types of Language are influenced by various contingencies that affect the manifestations of the *Languages of Grief*. The model presented in Figure 1 is not about the content of the communication, but rather how the communication is presented.

### 4.1 Modes of Expression

Modes of Expression include verbal responses (written or oral); nonverbal responses (silent or reflective); physical responses (somatic or expressions); and physical activities (rituals or objects). Examining grief in children provides a paradigm for understanding the Modes of Expression of grief because the differences in a child's expressions over time are distinct. For example, a toddler may manifest clinginess, regression to a previous behavior like thumb-sucking, or crying (physical responses) as responses to grief (D'Antonio, 2011). Parallel responses in a school-aged child may include trouble sleeping (physical response), wearing an article of the deceased's clothing (physical activity), or fear of being abandoned (nonverbal). The changes that occur in a child's manifestations of grief during development provide a prelude to his or her adult responses. In the model of the *Languages of Grief*, a particular Mode of Expression was not conferred with privileged or pre-eminent status. The Modes of Expression are explicated below.

#### 4.1.1 Verbal responses

Verbal responses are those that communicate one's reaction to bereavement with the use of words. The bereaved may express their grief using different spoken languages and in some languages there may be no direct translation of the word grief such as in Portuguese where the term *suffering* encompasses the concept of grief. Nonetheless, the concept of grief is articulated verbally in sounds that can be both heard and comprehended by others familiar with the language.

#### 4.1.2 Nonverbal responses

Nonverbal responses are thoughts or reflections kept to oneself. Nonverbal expressions may be silent or reflective, i.e. thinking about the experience. They also may be expressed in another format at a later time. The absence of an outward expression of emotion is also part of the *Languages of Grief*.

#### 4.1.3 Physical responses

Physical responses are composed of physical signs, bodily expressions, and sensual aspects such as seeing and hearing. The manner in which the head is held (body language) is an example of a physical response, as is weeping. Sobbing, sighing, sudden, intense emotion, or other somatic, physical responses typically occur without intention.

#### 4.1.4 Physical activities

Physical activities are intentional expressions that involve action or objects. Physical activities are exemplified by attending funerals, planning and holding memorial services, or by *sitting Shiva* in the Jewish tradition or other expressions of respect for the dead and the bereaved. These physical activities may have both public and private components. The purpose of expression may be to satisfy social conventions, communicate with those nearby in order to connect with them, and/or to define the dead person for oneself. This is achieved by such activities as public or private ceremonies, visits, correspondence, and inscriptions in memorial books. Gilroy and Johnson (2004) note that physical expression through activities such as play is one of the themes that emerged in observing grieving children. Another example of a physical activity is to make a sudden change in one's lifestyle. For example, suddenly selling one's home and moving far away is an accommodation made by a griever to their bereaved situation. These dimensions of Modes of Expression are enriched by an analysis of Types of Language.

### 4.2 Types of Language

Types of Language consist of four subsets – Narrative (story telling), Symbolism (representation), Metaphor (figurative), and Analysis (concretizing). These are the Types of Language used by the bereaved to express their grief.

#### 4.2.1 Narrative

Narrative is synonymous with storytelling and is a familiar form of communication for the bereaved who tell and retell the story of the death of their loved one (Hedtke, 2002). The narrative is framed by the religious, spiritual, and cultural beliefs of the individual and his/her closest relatives and friends. Hedtke (2002) emphasizes the importance of narrative in working with dying persons and their loved ones as does Berzoff (2006), who considers it essential for those who want to transform grief into political action. An example of the latter is the establishment of a foundation such as the Komen Foundation founded by the sister of a woman who died of breast cancer. The Komen Foundation raises money for cancer research. A family member, friend, or grief counselor uniquely influences and co-creates a griever's narrative simply by listening (Gilbert, 2002). This idea of a present listener is an important element in understanding the model's representation of narrative.

The narrative form of language, when combined with *Modes of Expression*, results in distinctive approaches to the *Languages of Grief*. When narrative is expressed by physical activities, for example, one might participate in a memorial service by reading a eulogy. Narrative when expressed by verbal (written) activities is illustrated by maintaining a journal or by writing poetry (Cunningham, 2009). Finally, narrative combined with nonverbal (reflective) activity is exemplified by sitting in a favorite place and internally reflecting on the person who died.

#### 4.2.2 Symbolism

Symbolism represents the material, action, and performance manifestations of the *Languages of Grief*. Coleman (2010) observed that symbols are integral to a relationship and develop over the course of a relationship. Symbols may represent the relationship or an object of importance in the relationship. Symbolic language involves action or activity that represents a personal interpretation of a loss. The spouse of a fisherman might scatter his ashes at sea, a physical activity with symbolic meaning. Mothers who have experienced the death of their babies often describe an aching sensation in their arms, a symbolic physical response, as the aching reminds them of the baby they hoped to cradle. A child whose mother died may look at the nighttime sky and say, “Mummy is a new star in the sky” (verbal symbolic). The newlywed whose wife died in an automobile accident may request that the song the couple referred to as “our song” be played at her funeral, a nonverbal symbol of their relationship. Another form of symbolic representation is the creation of a shrine where some public tragedy has occurred by the placement of flowers or memorial items such as those placed at the side of the road where an automobile accident resulting in a death occurred (Jorgenson-Earp & Lanzilotti, 1998), or the construction of a collage (Rogers, 2007) that depicts images of personal or symbolic importance to the deceased.

#### 4.2.3 Metaphor

A metaphor is figurative language depicting one phenomenon in terms of another. Metaphors are used to understand and describe a personal reality. Kenney (2002) described the use of metaphor as a means to present oneself or to express what seems inexpressible. Metaphors may be particularly helpful in addressing taboo subjects (Young, 2007). Being heartbroken or having a broken heart is metaphorical language to describe the sensation in one's chest of intense grief (metaphor and physical response). A grieving friend may create a jigsaw puzzle from the photo of a butterfly, a metaphor for her dead friend who danced “like a butterfly”; although the pieces of the puzzle can be reconstructed, the scars of separation remain.

The father of a 4-year-old who died of cancer uses metaphor when writing in his blog that he pictured his daughter as a sparkling crystal. A physical metaphor is exemplified by the bereaved wife who neglects self-care to reflect her grief or a child who considered the loss of a balloon as a metaphor for the death of her father (Moore, 1988). Another example is a cemetery in England overlooking the seaside with gravestones with no words from the wearing of the wind, salt, and weather. These gravestones have become works of art, a metaphor for grief itself – the elements of which are expressed in various combinations and may be transformed over time. Given the lack of inscription, the reader is free to interpret the gravestone's meaning.

Nadeau (2006) suggests that metaphors are a means of negotiating reality while Czechmeister (1994) emphasizes the role of metaphor in understanding a phenomenon, such as a nurse's understanding of a patient's illness. Metaphors provide a means to both cognitively and emotionally express grief, and are “basic to creative thought” (Czechmeister, 1994, p. 1232). Hospice professionals use metaphors to describe the containment of emotion as a way of expressing both feeling burdened and drained from repeatedly being with people who die (Froggatt, 1998). They speak of being a “sponge” and consciously distancing themselves from their emotions by stepping back, switching off, and developing a veneer (Froggatt, 1998, p. 335). Finally, Moules, Simonson, Prins, Angus, and Bell (2004) suggest the metaphor of grief as a houseguest that arrives without invitation, infiltrating all aspects of lives, families, relationships, and health. In this insidious infiltration, grief has the potential to take over lives and relationships and the more the effort is made to force it out, the more intrusive it becomes:

If, however, room is made for this houseguest, its presence becomes expected at times, its comings and goings are not surprises, its intrusions not unanticipated. In time, its presence even becomes welcome as something familiar … its very absence and presence serves to sustain a mutable, evolving, sometimes intermittent, but lifelong relationship with the loss. (Moules et al., 2004, p. 104)

This metaphor of the expected houseguest is congruent with the theme of continuing bonds with the deceased (Klass, Silverman, & Nickman, 1996).

#### 4.2.4 Analysis

Of the four basic uses of language, analysis refers to taking ideas about the death of an individual and identifying which ideas may be useful for some purpose. An example of this is conjecturing that some family members or friends may be more helpful and available (for assistance with practical tasks and emotionally) to the griever than others. Drawing a timeline of what the griever knew before the death that differs from what is known after the death is an analytic tool that is especially useful in the case of a sudden death. This approach can be used by the griever who is overwhelmed by the idea that s/he should have known or should have done more to save the life of the deceased (Wrenn, 1999).

Another illustration of moving one's grief into the realm of language is to ponder the comment by a “well-meaning” acquaintance that someone will eventually come along to take the place of the person who died; a comment that may or may not be welcomed or considered helpful. In the course of analyzing and making concrete ideas that are helpful to coping with the event with which the griever is struggling, the process may be transformed into: (1) the verbal realm by writing or speaking to others about these ideas; (2) silence by simply reflecting on the meaning of these ideas; (3) physical activity; or (4) a physical response.

An example of each of these transformations in the realm of analyzing (concretizing) is a physical response such as increased blood pressure or anxiety when thinking of some aspect of a death or a movement toward a physical activity such as gathering pictures of the deceased to display at the funeral or memorial service. A nonverbal response is a continuous mulling over of an idea in a silent or reflective way, keeping thoughts to oneself. Baddeley and Singer (2009) situate silence in the larger context of autobiographical memory. They note that silence about memory, “unspoken memory” (Baddeley & Singer, 2009, p. 198), influences and is influenced by both individual and family narrative. The authors frame silence as a social process, a way of building a narrative identity and coping with a death. Silence may be loss-oriented or restoration-oriented (Stroebe & Schut, 1999). In writing in a diary or a journal, one would move toward a verbal expression of the thoughts and ideas being analyzed.

### 4.3 Contingent Factors

A variety of Modes of Expression and Types of Language are available to those who are grieving by which they can express themselves, consciously or unconsciously. There are, however, certain Contingent Factors that alter expression that a skilled listener will discern beyond what is said or expressed. Contingent Factors can be schematized into the internal (personal history and emotions), the interpersonal (social support and expectations of the self and other), and the external (cultural expectations and public expectations). For the individual, many Contingent Factors will remain largely the same over the course of a lifetime, although there may be variations with time and stage of life.

#### 4.3.1 Internal factors

The Internal Contingent Factors that affect the manifestations of grief or content of communications may include personal history, education, previous bereavement, emotional intelligence, personal theology/spirituality, financial issues, linguistic ability, and life expectancy. Emotions are Internal Contingent Factors that will influence individual expression (e.g. anger, depression, and vulnerability). These and other emotions may continue to manifest themselves to the point that to some listeners they seem integral to the person rather than a manifestation of grief.

#### 4.3.2 Interpersonal factors

The first set of Interpersonal Contingent Factors is about social support including the number and depth of previous friendships; relationships in the family and the community; available networks of interest, activity, or belief; and the variety of listening types – and people – available to the griever. The second group of Interpersonal Contingent Factors is the set of expectations held by the griever – of being heard by others; of having the energy to communicate; of boring or embarrassing others; of embarrassing themselves by crying; and other reasons to self-censor.

#### 4.3.3 External factors

External Contingent Factors include cultural expectations such as the official languages of state, religion, and sub-group; the authoritative discourse of physicians and other professionals with their technical languages; power relationships with professionals; and conventions in ritual settings and the nature of the death. For example, the term “closure” is a well-known, yet less useful, way of defining outcomes of grieving, whereas the phrase “continuing bonds” acknowledges the actual experience of many grievers. Both terms are examples of appropriated language or language taken from the professional sphere. Rosenblatt (2008) argues that a post-modern approach would entail no single concept being used to define experience or what is desirable post bereavement.

The importance of working with the bereaved, so as to maintain connection, is stressed by Cowdery and Andrews (2005). This connection is challenged when the language of professional communication is significantly different from the language of experience. A further challenge is the grievers' understanding of public expectations that in turn affect their expression of grief: the discomfort of crying in public places; what is considered appropriate public display of emotion; the length of time spent grieving and how soon “normality” is expected. Hendry (2009) presents the example of constrained languages and manifestations of grief used by those who are incarcerated and experience the death of a loved one. A prisoner may be told of the death of a loved one but fear expressing strong emotions for concern about being placed on suicide precautions.

An important Contingent Factor that influences the Types of Language and the Modes of Expression exhibited is the nature of the death itself. Was it expected or not? Was it violent or not? Was it the result of disease or “old age”? Was the individual a young person? Miscarriages, birth of a stillborn, death of a child engaged in sports, the death of a person of 100 years of age, as well as other types of death all have an influence on how grief is expressed and by whom.

## 5. Exemplars: acknowledgment of the deceased

Gravestone inscriptions are exemplars of some of what has been explored in this paper. These inscriptions may be prescribed, symbolic (angel), or a deeply personal narrative (e.g. “kept apart in life, together in death” for a gay couple). Other examples include: “It was hard to give thee up but thy will O God be done”; “The life that touches the hearts of others lives on forever”; and “She loved rainbows.” These phrases on the gravestones depict the sentiments of the grievers about the deceased (or those associated with memorialization) or their perspective on what is considered appropriate. Phrases on gravestones are meaningful to those who arrange for the memorial stone. They may be prescribed by religion or custom or may be an expression of individuality. Inscriptions may also be determined by individuals closer to or further removed from the deceased including:

- The deceased who left specific wishes and instructions.
- The centrally bereaved.
- The marginally bereaved supporting the centrally bereaved.
- The supportive community.
- Professionals related to death – clergy.
- Professionals related to disposition – funeral directors.
- Professionals related to grieving processes – some of the above, counselors, psychologists.
- Professionals related to memorialization – clergy, masons, superintendents of cemeteries, and crematoria.

A gravestone is a way of honoring (or at the least acknowledging) the life and death of an individual. This expression is static. A more active expression is communication with the deceased.

## 6. Communication with the deceased

In other venues, the griever may ask the deceased to intercede on his/her behalf for divine intervention. The deceased becomes the intermediary. Most clearly allied to the “continuing bonds” theory is the expectation that the dead will act in some way. Some prayers ask for divine intervention to promote communication between the living and the dead. The numerous messages written directly to the dead include appeals to the dead to perform some action. The dead are generally believed to be among the other dead: caring for the other dead, watching over the living, guiding the living, and communicating with the living. Such beliefs exemplify the reciprocity of a continued relationship, one that provides benefits and succor to the griever.

Different *Languages of Grief* may be used in messages written to the dead such as those found in a book in a hospice chapel. These messages include the perception of continuing connection such as: “We've just come back from skiing in France, but I know you were here with us all”; and:

Dear J, I suppose yet again I'm writing things I found it difficult to say when you were alive. I miss you so much and there is a huge hole where you were. You were so cold when I kissed you good-bye. (D. Head, Personal communication)

The Internet has provided a vehicle for writing messages to the dead and opening the memorial book for those who attend a funeral and to family and friends separated by distance (Mitchell, Stephenson, Cadell, & Macdonald, 2012). Unless closed, these virtual memorials also provide a means for those who are strangers and are browsing the Internet or who have similar sites to visit and express their condolences. *Facebook* also provides a vehicle for “communicating” with the dead and a means of expressing continuing connection. An example is:

Hi my dear daughter. Today you gave me a beautiful gift for being 59 years married … . You brought my mother to me, and you too dear … . It was great to see you and know you are with those you love.

As is obvious, these communications are not dissimilar to those found in the book in the small hospice chapel and are a vivid and public expression of grieving and continuing bonds with the deceased.

## 7. Implications for professionals

Professionals use a theoretical language to describe the process of grieving. The focus on this language with the implications of stages changes the focus from the bereaved and their expression of grieving to one of the processes. The focus on process distracts the professional from attending to the expressions of grief whether verbal, nonverbal, a physical response, or a physical activity. Attention to the Modes of Expression, Types of Language, and Contingent Factors will provide the professional with a richer understanding of grieving, a first step in providing appropriate support to the bereaved.

## 8. Conclusions

The *Languages of Grief* comprise a complex interaction of Types of Language, Modes of Expression, and Contingent Factors. A different language may be used by the same person on different occasions at different times of his/her life. Understanding the *Languages of Grief* requires careful observation and must be appreciated in the context of relational aspects and culture (Contingent Factors). This model also contributes to clinical understanding and fruitful exploration of the specific languages an individual uses to express his or her grief. Clearly, the expressions used will be reflective of the numerous factors that influence behavior in a given context.

The purpose of professional involvement is to allow the griever to engage in a discourse that provides the bereaved with satisfactory meanings of his/her grief experiences. In order to do so, the professional must be respectful of the griever's use of Modes of Expression and Types of Language taking into consideration Contingent Factors. The model presented here may help professionals do so.

The model of *Languages of Grief* generates potential research questions. Are differences in the resolution of grief related to the Modes of Expression utilized? How are Contingent Factors related to the use of *Languages of Grief*? Does online or face-to-face group support modify one's personal language of grief? How are the *Languages of Grief* expressed in the mass media? Investigation of these questions may enhance our understanding of grief and the languages used in its expression.

In summary, grievers have different ways of expressing their grief. All of the ways in which this communication is presented may be described as the *Languages of Grief*. The model of the *Languages of Grief* provides a framework for considering the varied manifestations of grief. These manifestations are not mutually exclusive and are presumed to occur both simultaneously and sequentially but not in a prescribed order.

The model of the *Languages of Grief* is not about the content of the communication, but rather the dimensions of the communication and the languages in which the communication is presented. This offers a range of modalities for those who find themselves “stuck” or who wish to express themselves in a variety of ways. The model represents a cross-sectional approach and should be viewed as representing one moment in time and therefore is not a longitudinal representation.

The *Languages of Grief* presented in this model provides a framework for application to discrete occurrences with the goal of understanding grief from the perspective of the bereaved. The authors of the model have focused on the very practical goal of identifying expressive modes, language types, and contingencies that make grief more understandable for the griever and those bearing witness.
